# The integrated analysis of SIRT family expression, prognostic value, and potential implications in childhood acute lymphoblastic leukemia

**DOI:** 10.3389/fonc.2025.1685249

**Published:** 2025-10-15

**Authors:** Xusan Xu, Zhendong Wang, Xiaoxia Wang, Wensen Zhang, Zhengqiang Luo, Xiaomei Zheng, Ronghua Pan, Ying Fu, Yajun Wang, Guochun Huang, Riling Chen, Guoda Ma

**Affiliations:** ^1^ Institute of Pediatric Hemato-oncology, Shunde Women and Children’s Hospital, Guangdong Medical University, Foshan, China; ^2^ Department of Pediatrics, Shunde Women and Children’s Hospital, Guangdong Medical University, Foshan, China; ^3^ Department of Neurology, Longjiang Hospital, Foshan, China; ^4^ Institute of Pediatrics, Shunde Women and Children’s Hospital, Guangdong Medical University, Foshan, China

**Keywords:** childhood acute lymphoblastic leukemia, SIRT1, overall survival, prognostic factor, drug sensitivity

## Abstract

**Background:**

Acute lymphoblastic leukemia (ALL) is a rapidly progressive hematological malignancy caused by the dysregulated proliferation and abnormal differentiation or differentiation block of lymphoid precursors. The sirtuin family, as a highly conserved class of protein deacetylases dependent on NAD^+^, has been widely reported in leukemia. However, there has been no research on the prognostic value and molecular functions of the sirtuin protein family in pediatric ALL.

**Methods:**

In this study, we employed the Therapeutically Applicable Research to Generate Effective Treatments (TARGET), Genotype-Tissue Expression (GTEx), Encyclopedia of RNA Interactomes (ENCORI), Cancer Therapeutics Response Portal (CTRP), and STRING databases as well as R language to explore and visualize the role of the sirtuin family in childhood ALL. The receiver operating characteristic (ROC) curve was performed to investigate their diagnostic value, while the Kaplan–Meier survival curve and Cox regression analysis were utilized to test their prognostic value. Additionally, we conducted Pearson correlation analysis to explore the association between sirtuin family mRNA expression and DNA methylation.

**Results:**

Our results indicate that sirtuin family mRNA expression is dysregulated in pediatric ALL. The ROC curve revealed that SIRT1 and SIRT4 expression is highly sensitive and specific in diagnosing childhood ALL (AUC > 85.0%, *p* < 0.001). While higher SIRT1, SIRT4, SIRT5, and SIRT7 expression was related to higher event-free survival rate and overall survival (OS) rate, higher SIRT2 expression was associated with lower event-free survival rate and rate in childhood ALL (*p* < 0.05). Moreover, Cox regression and nomogram analyses suggested that SIRT1 mRNA expression is an independent factor for pediatric ALL. Subtype analysis revealed that SIRT1 primarily functions in B-cell precursor ALL (B-ALL). Furthermore, SIRT1 is involved in various RNA splicing and acetyltransferase complex in B-ALL. The data from the CTRP database and the Cell Counting Kit-8 (CCK-8) experiment suggested that SIRT1 increased the sensitivity of B-ALL cell lines to vincristine. *In vitro* experiments demonstrated that SIRT1 inhibits invasion activity in B-ALL cell lines (NALM6 and REH).

**Conclusions:**

SIRT1 represents a potential prognostic biomarker and therapeutic target in childhood B-ALL.

## Introduction

Acute lymphoblastic leukemia (ALL) is the most significant malignant tumor affecting children's health, and its incidence has been on the rise in recent years (1), posing a severe threat to the health of children worldwide. The current treatments for ALL have greatly improved the survival rates of children with the disease, but 10%–20% of them still have poor prognoses (2). Moreover, drug-induced toxicities constitute another major challenge to the prognosis of patients with ALL. The cornerstone drug vincristine, for example, leads to peripheral neuropathy in up to 78% of patients (3). Therefore, finding new diagnostic biomarkers and therapeutic targets for ALL to facilitate earlier intervention and reduce drug dosage is of great significance for further improving the treatment outcomes.

The sirtuin (SIRT) protein family belongs to class III histone deacetylases. Currently, seven SIRT family members have been identified in humans: SIRT1–SIRT7 (4). SIRT proteins regulate gene transcription and expression by deacetylating histones and a wide range of non-histone substrates, such as p53, fork head box protein O (FOXO), and signal transducer and activator of transcription 3 (STAT3), thereby participating in various biological processes, including cellular homeostasis, inflammation, aging, circadian rhythms (5), and hematopoietic homeostasis (6). Previous studies have reported dysregulation expression of the SIRT family in various types of leukemia (7). SIRT1 is overexpressed in patients with T-ALL (8). Compared to normal peripheral blood B cells, chronic lymphocyte leukemia (CLL) samples exhibit decreased SIRT4 but increased SIRT3, SIRT6, and SIRT7 (9). The exact role of the SIRT family in leukemia appears somewhat paradoxical. In T-ALL, SIRT1 drives proliferation by deacetylating KAT7 (8). In contrast, Lei et al. reported that an oncolytic vaccinia virus expressing Beclin-1 induced autophagy by upregulating SIRT1, which led to leukemia cell death (10). Furthermore, Dong and colleagues revealed that SIRT5 mediated the alleviation of 6-MP resistance in B-ALL by histidine through the desuccinylation of HINT1 (11). However, there are relatively few reports on the role of SIRT family members in childhood ALL. Further study on the SIRT family in pediatric ALL may help identify new therapeutic targets.

In this study, we selected the Therapeutically Applicable Research to Generate Effective Treatments (TARGET) database, which includes only pediatric tumor data, to preliminarily explore the expression profile, diagnostic value, prognostic value of the SIRT protein family, and the potential association between SIRT family mRNA expression and relapse, DNA methylation, and enriched pathways in pediatric ALL. In addition, we evaluated the impact of SIRT1 on the drug sensitivity, proliferation, and invasion of ALL *in vitro* models.

## Materials and methods

### Data source and processing

We achieved mRNA expression (mRNAseq technology: Illumina Hiseq 2000), DNA methylation, somatic mutation, copy number variation (CNV) data, and clinical information of childhood ALL from the TARGET database ALL cohort (https://portal.gdc.cancer.gov/analysis_page?app=Downloads). Inclusion criteria are as follows: Patients met the diagnostic criteria for ALL according to the World Health Organization (WHO) classification of tumors of hematopoietic and lymphoid tissues, and was risk-stratified and treated in accordance with the Children's Oncology Group (COG) clinical practice guidelines. Samples with ambiguous subtype or without RNA-seq data were excluded. After quality control and the application of inclusion criteria, the cohort included 480 childhood ALL, with the following subtypes: 210 B-ALL, 265 T-ALL, and 5 other types of ALL. A detailed summary of patient characteristics is provided in **Supplementary Table S1**. We also acquired mRNA-seq data (mRNAseq technology: Illumina Hiseq 2000) of blood samples from healthy controls from the Genotype-Tissue Expression (GTEx, http://gtexportal.org/) database. For mRNA-seq data, we removed low-expression genes (median counts < 10), converted counts to log2^(TPM + 1)^, normalized the data using the removeBatchEffect function from the limma R package v3.62.2, and finally used it for downstream analysis.

### Functional enrichment analysis

We uploaded the genes most correlated with SIRT1 mRNA expression in the B-ALL cohort for Gene Ontology (GO) analysis and Kyoto Encyclopedia of Genes and Genomes (KEGG) pathway (12) analysis via the R package clusterprofiler v4.12.6. The most correlated genes were ranked based on their FDR (FDR < 0.05) and *R* (|*R*| > 0.5). The top 500 ranked genes were selected for enrichment analysis to focus on the most robust and biologically relevant signals of SIRT1. The outcomes were shown in ascending order of gene ratio, showing the top 10. To validate the enriched results of biological process (BP), cellular component (CC), molecular function (MF), and signaling pathways mentioned above, Gene Set Enrichment Analysis (GSEA) was applied using the R package clusterprofiler v4.12.6 (R environment).

### The lncRNA–miRNA–mRNA network analysis

Given the pivotal role of post-transcriptional regulation in controlling SIRT1 abundance (13), we sought to identify long non-coding RNAs (lncRNAs) that may lead to aberrant SIRT1 expression in B-ALL by competing for microRNAs (miRNAs) that directly target SIRT1. First, remove lncRNAs and miRNAs with median counts < 10 in ALL. Then, Pearson correlation analysis was applied to gain the expression correlation between lncRNAs or miRNAs and SIRT1. For lncRNA-SIRT1, results where |*R*| > 0.3 and FDR < 0.05 were retained, and for miRNA-SIRT1, results where FDR < 0.05 were reserved. Finally, intersect these results with the lncRNA–miRNA and miRNA-SIRT1 data from the Encyclopedia of RNA Interactomes (ENCORI, https://starbase.sysu.edu.cn/index.php) (14) database. Additionally, the lncRNA–miRNA–mRNA network was drawn using Cytoscape software v3.10.3.

### The protein–protein interaction network analysis

We conducted protein–protein interaction (PPI) network analysis by uploading the top 500 genes (|*R*| > 0.5 and FDR < 0.05) most related with SIRT1 mRNA expression in the B-ALL cohort to the STRING v12.0 (https://cn.string-db.org/) database (organism: *Homo sapiens*, minimum interaction confidence score: 0.4). Import the results from STRING into Cytoscape software v3.10.3 and identify hub genes of the SIRT1 gene in B-ALL by calculating algorithms of the CytoNCA plugin (betweenness, closeness, degree, eigenvector, LAC, and network). Genes with all parameters higher than the median were considered core targets.

### Drug sensitivity analysis

Food and Drug Administration (FDA)-approved or clinically approved drugs were involved in drug sensitivity analysis. The IC_50_ (half-maximal inhibitory concentration) data of these drugs and SIRT1 mRNA expression data among different cancer cell lines were downloaded from the Cancer Therapeutics Response Portal (CTRP) database. Results where |*R*| > 0.3 and FDR < 0.05 were retained and were reserved.

### Cell culture and processing

The human ALL cell lines (NALM6 and REH) were purchased from the ATCC (American Type Culture Collection, Manassas, VA, USA). Both cell lines were confirmed using STR profiling. Both cell lines were cultured with 81% RPMI 1640 medium, 10% fetal bovine serum (FBS), and 1% penicillin/streptomycin in a 37°C incubator with 5% CO_2_.

SRT2104 was bought from Beyotime Biotechnology (Nanjing, Jiangsu, China). Subsequent experiments used SRT2104 with a final concentration of 4 μM to activate the protein expression of Sirt1 in this study. Vincristine sulfate (0.1 μM; MCE Company, USA) was applied in the drug sensitivity trial.

### Cell Counting Kit-8 assay

A total of 5,000 cells were prepared for drug susceptibility testing, and 2,000 cells were collected for cell proliferation assay. Cells were suspended in 100 μL of medium and seeded in a 96-well plate. The test was initiated after 24 h and lasted for three consecutive days (1, 2, and 3 days). To each well, 10 μL of Cell Counting Kit-8 (CCK-8) reagent was added. After 1 h at 37°C, the absorbance at 450 nm was tested using a microplate reader (BioTek, USA).

### Western blotting

The total protein of cells was extracted using RIPA lysis buffer and quantified using the BCA protein assay kit (Beyotime, China). The protein samples were then mixed with 4× loading buffer and heated at 100°C for 10 min. Subsequently, the samples were separated by sodium dodecyl sulfate–polyacrylamide gel electrophoresis (SDS-PAGE) and transferred onto a PVDF membrane. Thereafter, the membrane was blocked with 5% skim milk at room temperature for 1 h. Then, the blots were incubated overnight at 4°C with primary antibodies against Sirt1 (1:1,000, Abcam, ab189494), Bax (1:1,000, Abcam, ab7977), Bcl2 (1:250, Santa Cruz, sc7382), and β-tubulin (1:2,000, Proteintech, 10094-I-AP). After washing the membrane with PBST (PBS with 0.1% Tween-20), it was incubated with a secondary antibody (1:2,000) at room temperature for 45 min, followed by detection using the electro-chemiluminescence (ECL) method. The fluorescent signal was captured using a Bio-Rad imaging system (Bio-Rad, CA, USA). All raw data are displayed in the Supplementary Materials (**Supplementary Figure S1**).

### Transwell invasion assay

Transwell invasion assays were conducted using 24-well transwells (8 μm pore size, Corning, USA). The upper chamber of the transwell chamber was pre-coated with Matrigel matrix glue (Corning Company, USA; matrix glue: serum-free medium = 1:4). In the upper chamber, a total of 25,000 cells were seeded in 200 μL of serum-free medium. The lower chamber was filled with 750 μL of complete medium containing 10% FBS to act as a chemoattractant. After 48 h at 37 °C, the cells in the lower chamber were collected and calculated with a cell counter.

### Real-time quantitative polymerase chain reaction

Total RNA of cells was extracted using the TRIzol reagent (Gibco Company, USA), followed by reverse transcription into cDNA using a Prime Script RT Master Mix (Vazyme Company, China). Subsequently, quantitative polymerase chain reaction (qPCR) was conducted using a 2× SYBR Green qPCR Mix (Vazyme Company, China) to detect the levels of the targeted mRNA, and GAPDH was applied as the reference gene. The sequence of primers used in this research is displayed in **Supplementary Table S2**.

### Statistical analysis

The statistical analyses and visualization mentioned above were performed using R (version 4.4.1) and SPSS 21.0 software. An independent-samples *t*-test was used to compare the two groups. Pearson correlation analysis was conducted to assess the correlation between SIRT expression and the DNA methylation level in the ALL cohort. Receiver operating characteristic (ROC) curve analysis was used to access the diagnostic value. Cox regression analysis and Kaplan–Meier (K-M) curve (the cutoff for K-M survival analysis was determined by the median mRNA expression of SIRT family) were applied to evaluate the prognostic value. A *p*-value < 0.05 was considered statistically significant.

## Results

### SIRT family mRNA expression profiles in ALL

Compared to normal blood samples from the GTEx database, the mRNA expression of the SIRT family was dysregulated in ALL samples from the TARGET database when the mRNA expression of SIRT1, SIRT3, SIRT4, SIRT5, and SIRT6 was significantly increased, and the mRNA expression of SIRT2 and SIRT7 was decreased (**Supplementary Figure S1A**).

### The diagnostic value of SIRT family mRNA expression in ALL

To evaluate the diagnostic value of SIRT family mRNA expression for ALL disease or relapse, we conducted a ROC curve analysis. Our results suggested that SIRT1, SIRT3, SIRT4, and SIRT7 have the potential to serve as diagnostic biomarkers for ALL (**Supplementary Figures S1B–E**; **Supplementary Table S3**) and SIRT5 has the potential to serve as a diagnostic biomarker for ALL relapse (**Supplementary Figure S1F**; **Supplementary Table S4**) [area under the curve (AUC) > 0.70, *p* < 0.001].

### The association between SIRT family mRNA expression and ALL patient's prognosis

The K-M survival curves revealed that the higher mRNA expression of SIRT1, SIRT4, SIRT5, or SIRT7 has a longer event-free survival time in ALL, but the event-free survival time was shorter in ALL with the higher mRNA expression of SIRT2 (*p* < 0.05) (**Supplementary Figures S2A-G**). To further confirm whether the mRNA expression level of the SIRT family members could be independent prognostic factors in ALL, both univariate and multivariate Cox regression analyses were applied. The results found that SIRT1 expression was an independent prognostic factor in ALL, including other known prognostic factors, such as TCF3–PBX1 fusion gene status and cell of origin of ALL (*p* < 0.05) (**Supplementary Table S5**). In addition, the nomogram was established based on univariate analysis (**Supplementary Figure S2H**), which was used to predict the 1-, 2-, 3-, 5-, and 10-year event-free survival in ALL. The C-index and calibration curve were conducted to validate the accuracy of the nomogram (C-index = 0.851) (**Supplementary Figure S2I**).

The K-M curves found that the higher mRNA expression of SIRT1, SIRT4, SIRT5, or SIRT7 has a better overall survival (OS) in ALL, but the higher mRNA expression of SIRT2 was related to worse OS in ALL (*p* < 0.05) (**Supplementary Figures S3A–G**). Furthermore, univariate and multivariate Cox regression analysis revealed that mRNA expression of SIRT1 and SIRT5 and the status of the TCF3–PBX1 fusion gene were independent prognostic factors in ALL for OS (*p* < 0.05) (**Supplementary Table S6**). The nomogram was also built (**Supplementary Figure S3H**), and the C-index (C-index = 0.927) and calibration curve showed that the nomogram can predict the 1-, 2-, 3-, 5-, and 10-year OS in ALL very well (**Supplementary Figure S3I**).

### Copy number variation and DNA methylation analysis of the SIRT family in ALL

To investigate the underlying causes of SIRT family mRNA expression dysregulation in patients with ALL, we analyzed patients' somatic mutation, CNV, and DNA methylation data. No somatic mutation of the SIRT family was found in ALL. A few cases showed deletion copy of the SIRT family, which we did not include in the CNV analysis. We found that the mRNA expression of SIRT2, SIRT6, and SIRT7 was associated with CNV in ALL (**Supplementary Figures S4A–G**). DNA methylation analysis found that only the mRNA expression of SIRT2 was affected by the level of DNA methylation in ALL (*p* < 0.05, *R* = −0.30) (**Supplementary Figure S4H**).

### SIRT1 as an independent prognostic factor in B-ALL

**Figure 1:**
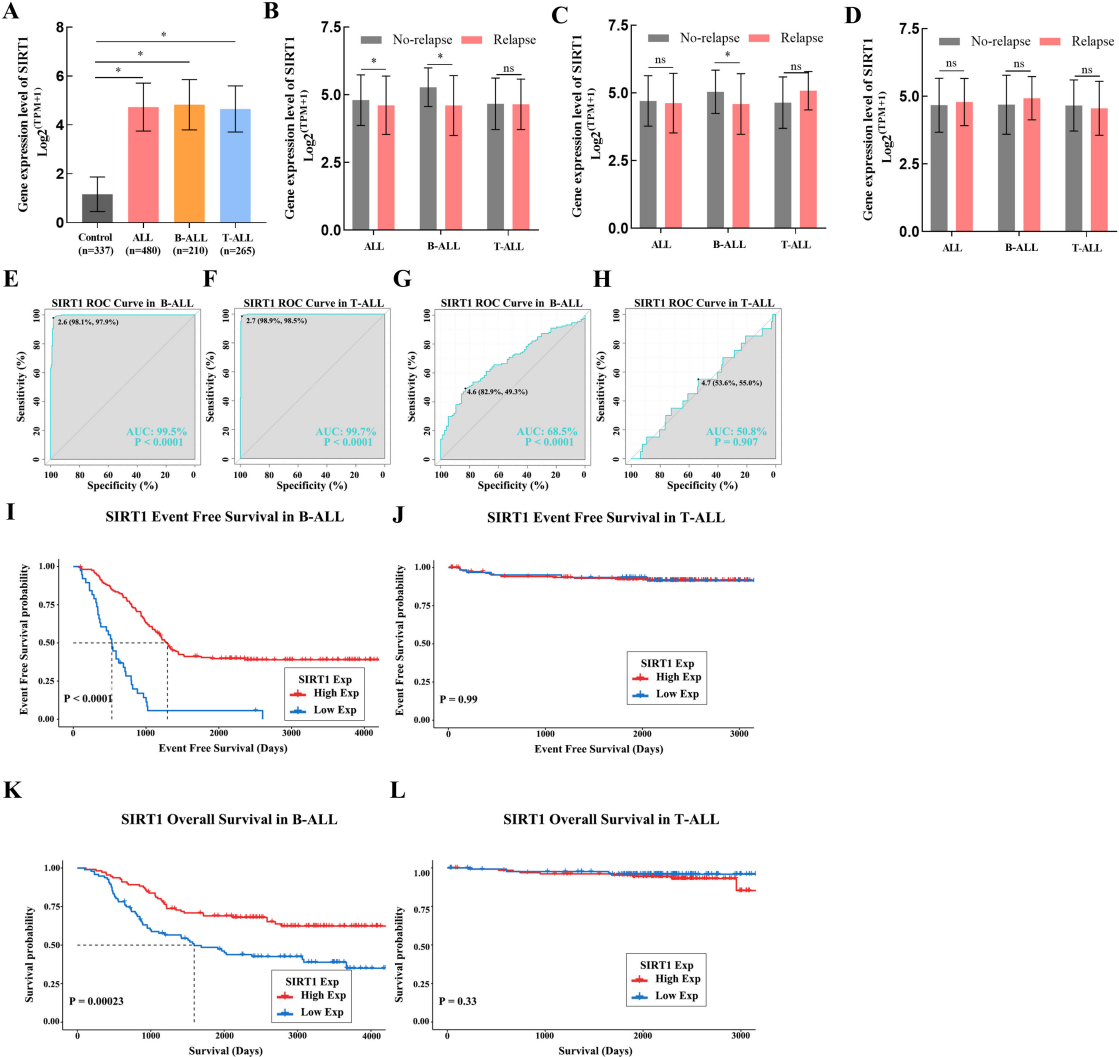
The subtype analysis of ALL based on SIRT1. **(A)** SIRT1 mRNA expression levels in normal and cancer blood samples of ALL or its subtype (B-ALL and T-ALL); SIRT1 mRNA expression in cancer blood samples of ALL or its subtype (B-ALL and T-ALL) with relapse **(B)**, with bone marrow of relapse **(C)**, and with central nervous system of relapse **(D, E, F)** The receiver operating characteristic (ROC) curve showed the expression specificity of SIRT1 in B-ALL and T-ALL. **(G, H)** The ROC curve showed the expression specificity of SIRT1 in B-ALL and T-ALL with relapse. **(I, J)** Kaplan–Meier **(K-M)** analysis of SIRT1 mRNA expression in B-ALL and T-ALL for event-free survival; **(K, L)** K-M analysis of SIRT1 mRNA expression in B-ALL and T-ALL OS. *p < 0.05; ***p < 0.001, AUC, area under the curve.

Because of the outstanding performance of SIRT1 in the onset and relapse of ALL, we conducted further subtype analysis of ALL based on SIRT1. We found that the expression of SIRT1 increased in both B-ALL and T-ALL (**Figure 1A**). Similar results were also observed when evaluating the diagnostic value of SIRT1 mRNA expression for B-ALL or T-ALL (**Figures 1E, F**). However, decreased mRNA expression of SIRT1 was only displayed in B-ALL with relapse or bone marrow of relapse, not in B-ALL with the central nervous system of relapse (**Figures 1B–D**). ROC curve analysis suggested that SIRT1 has the potential to serve as a diagnostic biomarker for B-ALL relapse but not T-ALL relapse (**Figures 1G; H**). Moreover, both K-M survival curves and Cox regression analysis revealed that the expression of SIRT1 was only related to the event-free survival and OS of B-ALL, but not T-ALL (**Figures 1I–L**, **Tables 1**, **2**).

**Table 1:** Univariate analysis of SIRT1 expression in ALL for event-free survival.

Variable	Univariate analysis	
	*P*-value	HR (95% CI)
ALL	**0.007**	0.801 (0.682, 0.941)
B-ALL	**4.08E-12**	0.546 (0.460, 0.648)
T-ALL	0.924	0.978 (0.619, 1.546)

CI, confidence interval; HR, hazard ratio. SIRT1 expression was included as a continuous variable in the model.

Significant (p < 0.05) results are shown in bold.

**Table 2:** Univariate analysis of SIRT1 expression in ALL for overall survival.

Variable	Univariate analysis	
	*P*-value	HR (95% CI)
ALL	**0.008**	0.773 (0.639, 0.936)
B-ALL	**3.19E-06**	0.633 (0.523, 0.768)
T-ALL	0.636	1.141 (0.660, 1.973)

CI, confidence interval; HR, hazard ratio. SIRT1 expression was included as a continuous variable in the model.

Significant (p < 0.05) results are shown in bold.

**Figure 2:**
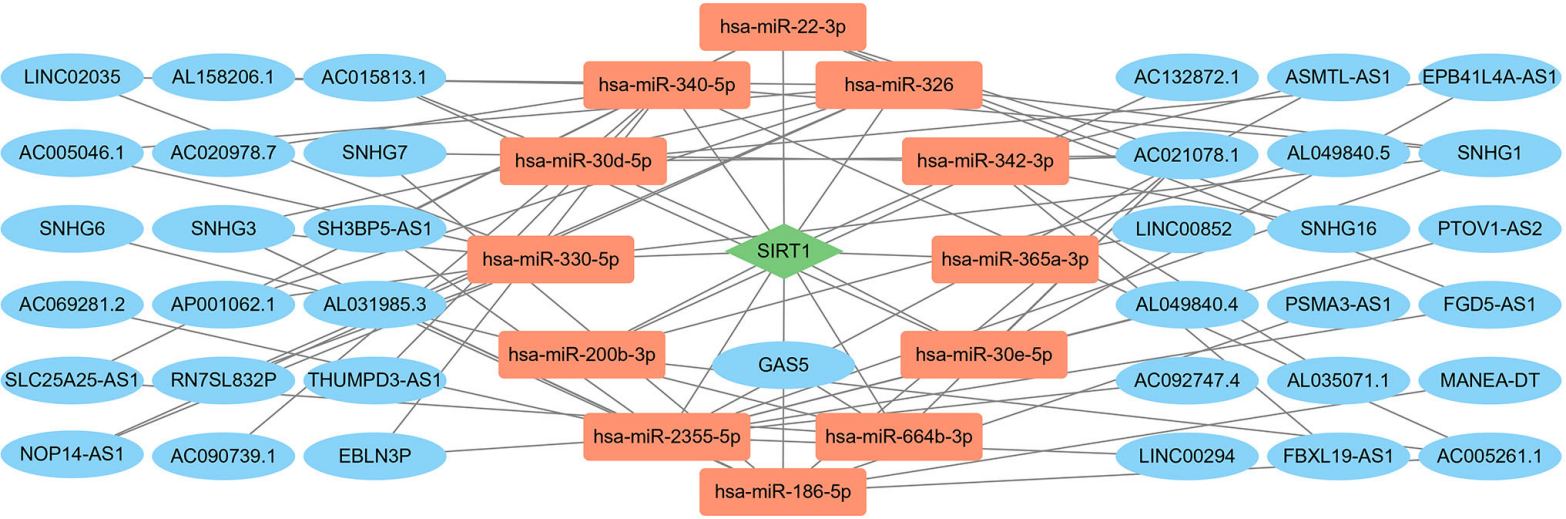
The LncRNA–miRNA–mRNA network of SIRT1 in B-ALL. Red nodes represent miRNAs; blue nodes represent lncRNAs; green nodes represent mRNAs.

The mRNA expression dysregulation may be driven by lncRNAs and miRNAs. Based on transcriptome data from the TARGET database B-ALL cohort and lncRNA–miRNA and miRNA–mRNA data from ENCORI, we constructed an lncRNA–miRNA–mRNA network of SIRT1 in B-ALL. As shown in **Figure 2**, 37 lncRNAs and 12 miRNAs were involved.

### Enrichment analyses and protein–protein interaction network of SIRT1 in B-ALL

**Figure 3:**
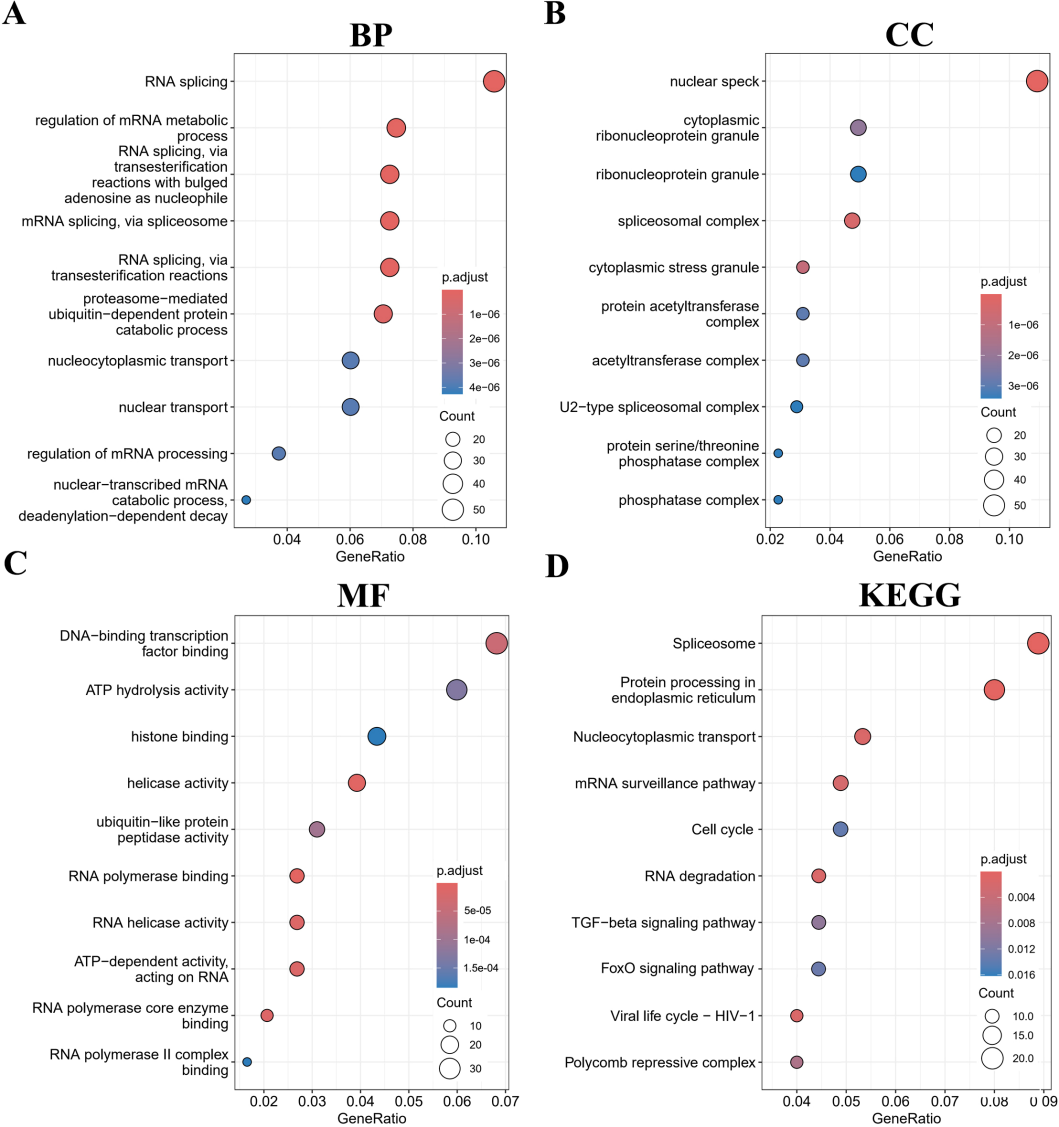
Gene Ontology (GO) and Kyoto Encyclopedia of Genes and Genomes (KEGG) (retrieved from http://www.kegg.jp/kegg/kegg1.html) enrichment analyses for SIRT1 in B-ALL. The top 10 pathways were enriched in the BP **(A)**, CC **(B)**, MF **(C)**, and KEGG **(D)**.

To investigate the biological functions of SIRT1 in B-ALL, the top 500 genes related to SIRT1 were acquired through Pearson correlation analysis based on the TARGET database B-ALL cohort. Then, GO and KEGG analyses were performed based on the above gene set. The most enriched BPs were mRNA processing, RNA splicing, positive regulation of transcription from RNA polymerase II promoter, mRNA splicing, via spliceosome, and positive regulation of transcription, DNA-templated (**Figure 3A**). Regarding CC, the most enriched CCs were nuclear speck, spliceosomal complex, cytoplasmic stress granule, cytoplasmic ribonucleoprotein granule, and protein acetyltransferase complex (**Figure 3B**). Furthermore, SIRT1’s most related MFs were RNA polymerase binding, helicase activity, RNA helicase activity, RNA polymerase core enzyme binding, and ATP-dependent activity, acting on RNA (**Figure 3C**). Additionally, KEGG analysis of SIRT1 enriched Protein processing in endoplasmic reticulum, mRNA surveillance, Cell cycle, and FoxO signaling pathway (**Figure 3D**), which aligns with the well-established roles of SIRT1 as a metabolic sensor and epigenetic regulator (15). This functional coherence confirms that our correlation-based selection strategy effectively captured biologically relevant targets and pathways potentially modulated by SIRT1 in B-ALL. Moreover, GSEA further confirmed the results of GO and KEGG analyses (**Supplementary Table S7**).

**Figure 4:**
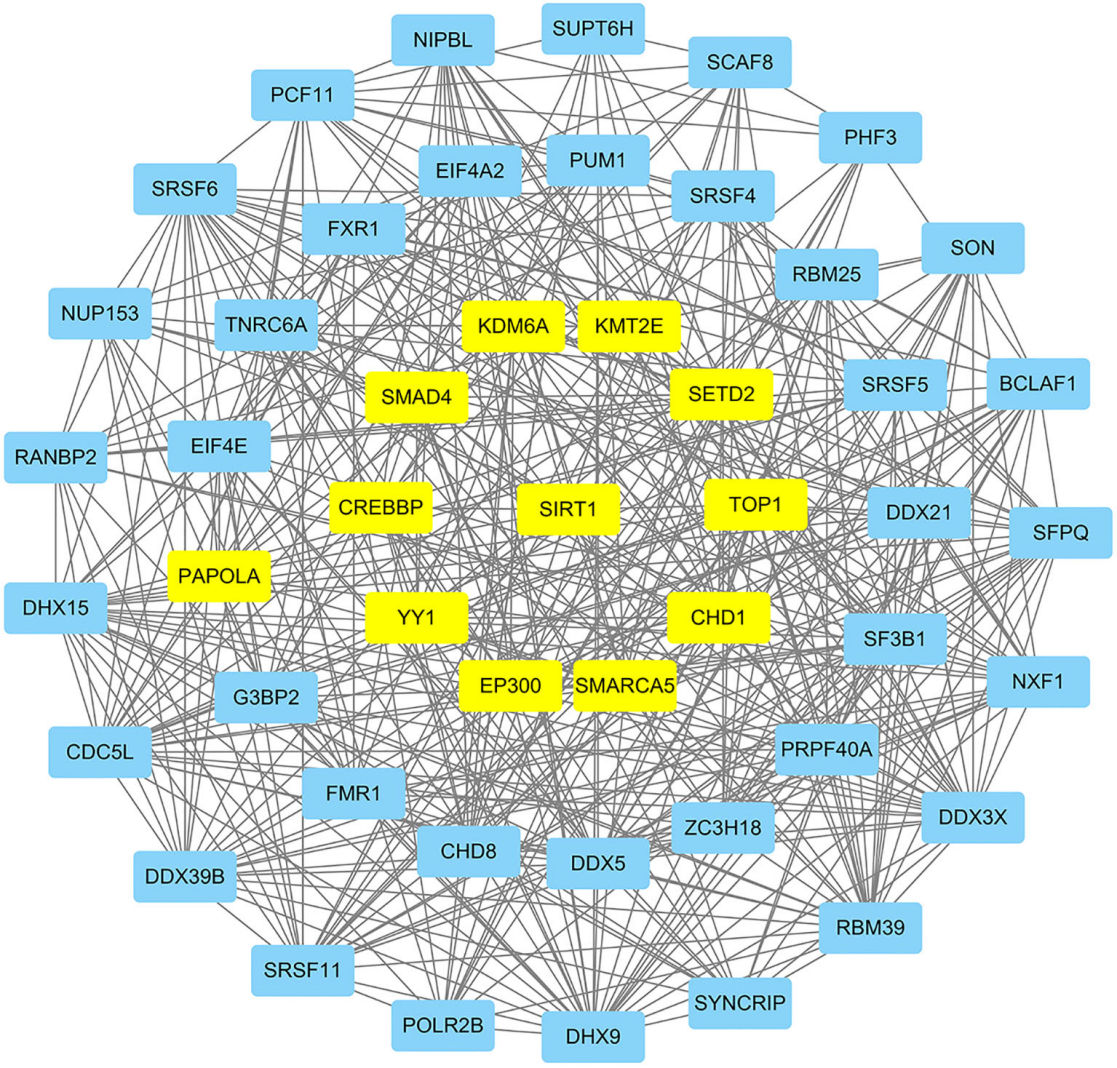
The protein–protein interaction (PPI) network of SIRT1 in B-ALL. Hub genes were highlighted in yellow.

Proteins affect the development and metastasis of tumors by interacting with other proteins. We performed a PPI network analysis of the top 500 genes related to SIRT1 using the STRING platform and algorithms of CytoNCA. As shown in **Figure 4**, we constructed a PPI network with SIRT1 as the core in B-ALL. This analysis identified 11 hub genes that interact with SIRT1, namely, SMAD4, CREBBP, PAPOLA, YY1, KDM6A, KMT2E, SETD2, TOP1, CHD1, SMARCA5, and EP300 (**Figure 4**).

### Drug sensitivity analysis of SIRT1

**Figure 5:**
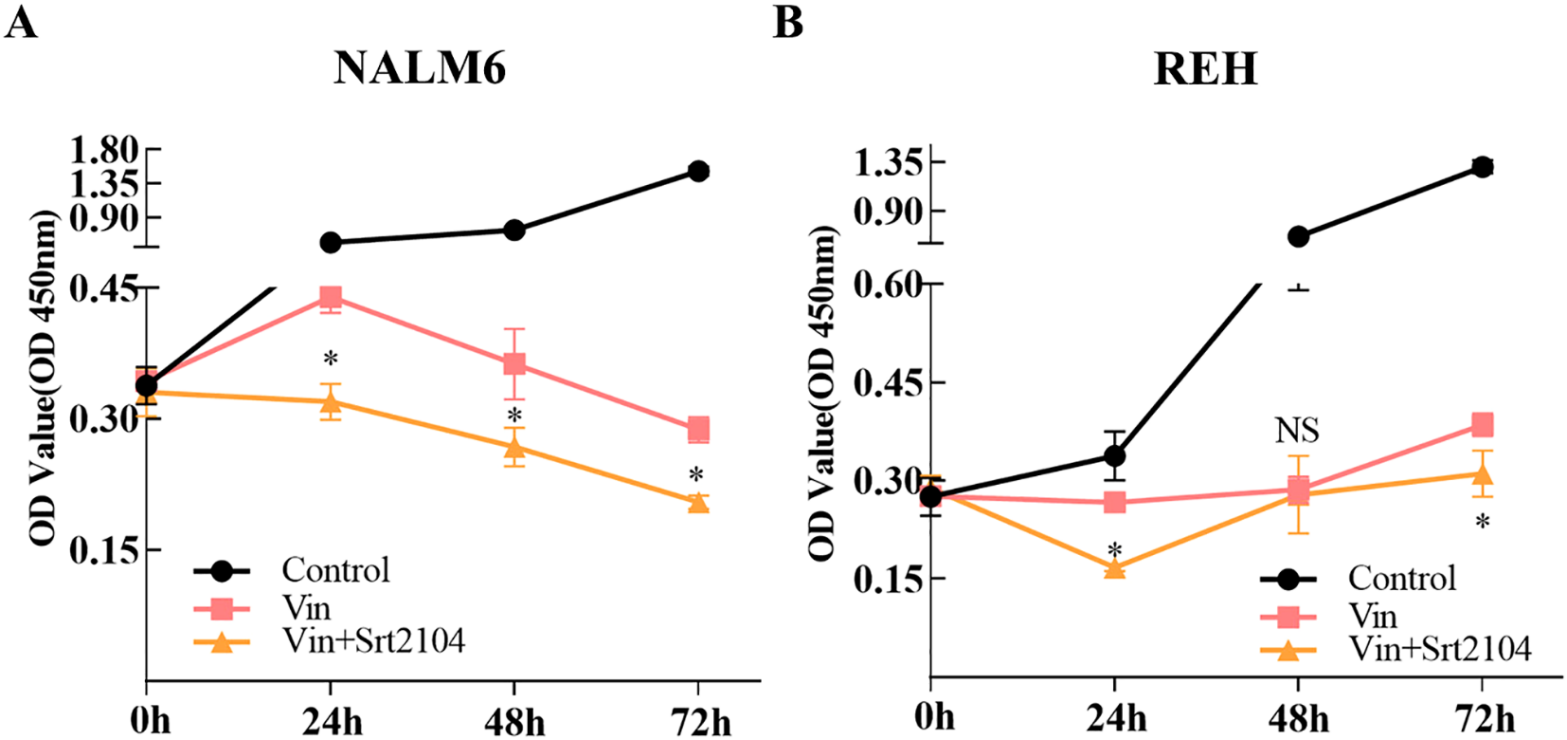
SIRT1 increased the sensitivity of ALL cell lines to vincristine. CCK-8 was performed to validate that SIRT1 affects the sensitivity of ALL cell lines NALM6 **(A)** and REH **(B)** to vincristine. *p < 0.05; ns: not significant.

The expression of SIRT1 is closely related to the drug resistance of various tumor cells. We collected data drug sensitivity information and SIRT1 mRNA expression data of various cancer cell lines from the CTRP database and found that the mRNA expression of SIRT1 negatively correlated with the IC_50_ of BI-2536, GSK461364, KW-2449, KX2-391, MK-1775, PX-12, axitinib, daporinad, indisulam, teniposide, tivantinib, and vincristine (|*R*| > 0.30, FDR < 0.05) (**Table 3**). Vincristine is a chemotherapy drug that is often used in combination with prednisone, daunorubicin, and asparaginase to treat ALL. *In vitro* experiments found that the SIRT1 activator SRT2104 further decreased the growth rate of B-ALL cell lines (NALM6 and REH) depressed by vincristine (**Figures 5A, B**).

**Table 3:** Drug sensitivity analysis of SIRT1.

Drugs	Cpd_status	Cor	FDR
BI-2536	Clinical	−0.306	9.08E−18
GSK461364	Clinical	−0.349	6.37E−22
KW-2449	Clinical	−0.309	1.74E−17
KX2-391	Clinical	−0.304	1.19E−16
MK-1775	Clinical	−0.330	4.20E−19
PX-12	Clinical	−0.341	6.86E−21
Axitinib	FDA	−0.308	4.41E−17
Daporinad	Clinical	−0.326	1.27E−15
Indisulam	Clinical	−0.311	1.18E−16
Teniposide	FDA	−0.352	3.87E−12
Tivantinib	Clinical	−0.329	7.33E−10
Vincristine	FDA	−0.303	1.30E−17

### SIRT1 suppresses migration and invasion activity of B-ALL cells

**Figure 6:**
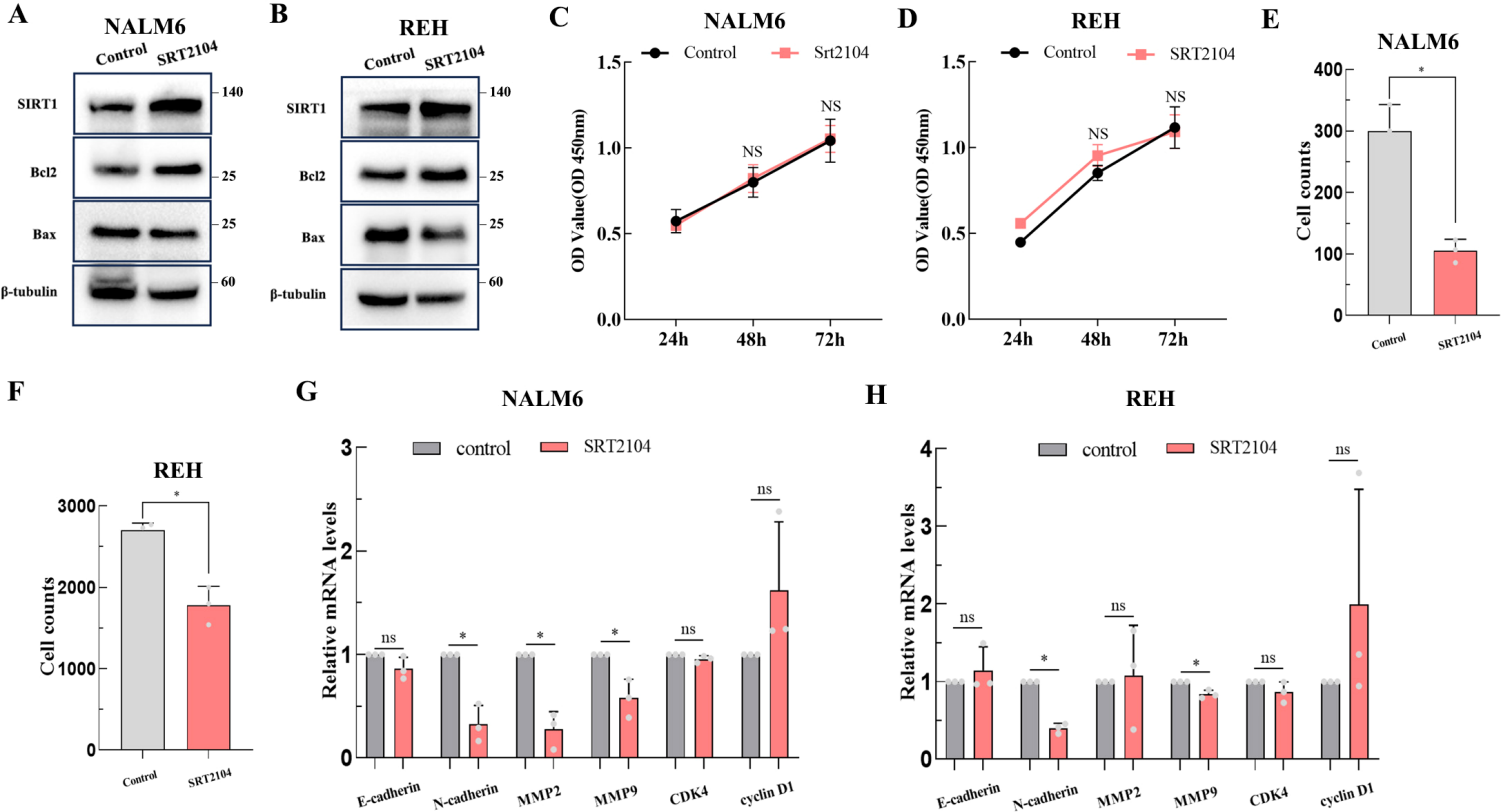
The biological functions of SIRT1 in ALL. The biological functions of SIRT1 on ALL cell lines were confirmed by apoptosis-related proteins **(A, B)** (the membranes were cropped at the indicated region specified in the Supplementary Materials), CCK-8 (**C, D)**, transwell invasion assay **(E, F)**, and qPCR **(G, H)**. *p < 0.05; ns: not significant.

SRT2104 is a highly selective Sirt1 activator. Therefore, we utilized SRT2104 to mediate Sirt1 protein expression in NALM6 and REH cell lines to observe any changes in the biological functions of ALL cells. **Figures 6A, B** show that SRT2104 significantly increased Sirt1 protein levels in NALM6 and REH cells. SIRT1 increased the apoptosis inhibitory protein Bcl-2 expression, and inhibition of pro-apoptotic protein Bax in NALM6 and REH cells (**Figures 6A, B**). The CCK-8 experiment conducted on the NALM6 and REH cell lines showed similar growth rates between the SRT2104 group and the control group (**Figures 6C, D**). The transwell invasion assay suggested that SIRT1 inhibits the invasion activity of ALL cells (**Figures 6E, F**). The qPCR results showed that SIRT1 decreased the expression of tumor invasion markers MMP2, MMP9, and N-cadherin in NALM6 cells, decreased the expression of N-cadherin and MMP9 in REH cells, but did not affect the cell cycle gene expression, such as CDK4 and cyclin D1 (**Figures 6F, G**).

## Discussion

Although the survival rate of childhood ALL has been increasing year by year, there is still a group of pediatric patients who are unresponsive to current treatment methods. Therefore, it is necessary to search for more sensitive diagnostic biomarkers and effective therapeutic targets for childhood ALL. The SIRT family, as deacetylases, has a broad range of substrates and is involved in various physiological and pathological processes such as inflammatory responses, oxidative stress, and aging (4, 16). Abnormal expression of SIRT family members has been observed in multiple types of cancers (17, 18) and is associated with tumor microenvironment (19) and tumor drug resistance (20). However, the role of the SIRT protein family in pediatric ALL has not been fully elucidated. Thus, in this study, we adopted bioinformatics approaches to access the potential roles of the SIRT protein family in childhood ALL from different perspectives, including mRNA expression profile, diagnostic value, prognostic value, drug sensitivity, lncRNA–miRNA–mRNA network, and PPI network, based on the TARGET, GTEx, ENCORI, CTRP, and STRING databases.

All members of the SIRT family exhibited dysregulation of mRNA expression in childhood ALL based on TARGET + GTEx databases (*p* < 0.05, **Supplementary Figure S1A**), which is consistent with previous data in adult ALL (21). This implied that the SIRT protein family may play an important role in the development and progression of ALL. DNA hypermethylation in the promoter region typically leads to gene silencing. The SIRT2 expression is significantly negatively correlated with its methylation levels (*p* < 0.05) (**Supplementary Figure S4**). This abnormal methylation pattern has also been reported in AML (22). However, it should be noted that since the correlation coefficient |*R*| < 0.5, the stability of this finding is relatively low. Further experimental validation is still required to determine whether the dysregulation of SIRT2 expression in ALL is indeed associated with methylation modification. The finding that no mutations were identified in the SIRT family in pediatric ALL from the TARGET database confirmed the conservation of the SIRT family (23). CNV can lead to genomic structural variations, thereby affecting gene expression and other biological characteristics. The correlation between the expression levels of SIRT2, SIRT6, and SIRT7 with copy number (*p* < 0.05) (**Supplementary Figure S4**) indicates that the dysregulation of SIRT2, SIRT6, and SIRT7 expression in childhood ALL may be related to CNV.

Relapse is one of the main reasons for poor prognosis in pediatric patients with ALL. ROC curve analysis indicated that SIRT5 mRNA levels are a relatively sensitive and specific marker for diagnosing childhood ALL relapse (AUC value = 70.8%) (**Supplementary Figure S1F**). Furthermore, K-M curves suggested that the mRNA expression levels of SIRT1, SIRT2, SIRT4, SIRT5, and SIRT7 were associated with event-free survival time in pediatric ALL (**Supplementary Figures S2A–G**). In particular, SIRT1 is an independent factor for childhood ALL relapse (**Supplementary Table S5**). A nomogram constructed with SIRT1, SIRT3, SIRT5, and clinical information can well predict the relapse of ALL in pediatric patients (**Supplementary Figure S2H, I**). These results suggest that the SIRT family, particularly SIRT1 and SIRT5, may play an important role in the relapse of pediatric ALL. The ROC curve also found that multiple members of the SIRT family are sensitive for diagnosing pediatric ALL, particularly SIRT1 (AUC value = 99.6%) (**Supplementary Figures S1B–E**). Furthermore, our results indicated that the SIRT family, in addition to SIRT3 and SIRT6, all possess value in predicting OS of pediatric ALL (**Supplementary Figures S3A–G**). Univariate and multivariate Cox regression analyses identified the expression of SIRT1 and SIRT5 as independent prognostic factors for pediatric patients with ALL (**Supplementary Table S6**). A nomogram constructed with SIRT1, SIRT5, and SIRT7, and clinical information can effectively predict the OS rate of pediatric patients with ALL (**Supplementary Figures S3H, I**). These results implied that the SIRT family, particularly SIRT1 and SIRT5, may influence the prognosis of pediatric ALL. Interestingly, a recent study reported that desuccinylation mediated by SIRT5 is an indispensable event for histidine combination therapy against 6-mercaptopurine resistance in pediatric patients with B-ALL (11). However, another study in AML found that inhibiting SIRT5 halted the progression of the disease (24). These findings highlighted the diverse and context-dependent impact of SIRT proteins in the pathogenesis of various types of leukemia. In all, the role of the SIRT protein family in pediatric ALL still needs further exploration in different racial or ethnic populations.

SIRT1 is one of the most studied members of the SIRT family. In this study, SIRT1 expression was found to be an independent prognostic factor for both relapse and OS, which prompted us to further explore whether SIRT1 functions in subtypes of ALL. Interestingly, the results suggested that SIRT1 only displayed significance in B-ALL (**Figure 1**; **Tables 1**, **2**), suggesting that SIRT1 may primarily function in B-ALL. Furthermore, compared to non-bone marrow relapse patients, SIRT1 mRNA expression was significantly reduced in bone marrow relapse B-ALL patients, while no difference was observed between non-CNS relapse and CNS relapse patients. These suggested that the decrease in SIRT1 expression may be related to the unique mechanisms of bone marrow relapse in B-ALL, and that SIRT1 is not a key factor in the pathogenesis of central nervous system relapse. Monitoring SIRT1 expression may help stratify patients at higher risk of bone marrow relapse, thus allowing for more targeted surveillance or preventive strategies, and modulating SIRT1 activity may be a potential therapeutic approach to prevent or treat bone marrow relapse. However, this requires further investigation, as SIRT1 may function as both oncogenes and tumor suppressors in cancers (25).

In recent years, a growing body of evidence has indicated that lncRNAs and miRNAs play important roles in the pathophysiology of hematological malignancies. Combining co-expression analysis and online databases, we constructed an lncRNA–miRNA–mRNA network for the SIRT1 in B-ALL (**Figure 2**). Philippe et al. found that miR-22-3p targets SIRT1, thereby decreasing cell viability and sensitizing AML cells to chemotherapy (26). LncRNAs GAS5 and SNHG7 have been reported to be involved in various cancers by indirectly regulating SIRT1 (27, 28). This regulatory network enriched our understanding of SIRT1 in B-ALL.

Enrichment analysis indicated that SIRT1 may be involved in the progression of B-ALL via impacting mRNA splicing (**Figure 3**; **Supplementary Table S7**). A previous study reported that SIRT1 suppressed splicing factor SC35-mediated tau exon 10 splicing through its deacetylation (29), providing evidence for SIRT1's indirect regulation of mRNA splicing. EP300, which co-regulates target proteins along with SIRT1, has recently been reported to increase the acetylation of the RNA splicing gene DDX5 promoter, promoting the progression of endometrial cancer (30). In the PPI network (**Figure 4**), EP300 and DDX5 were enriched. Whether SIRT1 is involved in the progression of B-ALL by co-regulating the acetylation status of DDX5 with EP300 requires further research to confirm.

Previous research indicated that SIRT1 activates chemoresistance in leukemia by deacetylating p53 in AML and p53, Ku70, Foxo1, and Hsp90 in CML (20). However, the results from Jiang et al. suggested that overexpression of SIRT1 sensitized ALL to the pan-histone deacetylase inhibitor panobinostat by inducing the mitochondria-related apoptosis pathway (31). Based on the data from the CTRP database, we found that the mRNA expression of SIRT1 is negatively correlated with the IC_50_ of vincristine (**Table 3**), a traditional chemotherapy agent used to treat ALL. SRT1720, a first-generation synthetic SIRT1 activator, was reported to significantly enhance vincristine-induced Ewing's sarcoma cell death (32). Moreover, our *in vitro* experiments further confirmed the sensitizing effect of SIRT1 on vincristine-induced B-ALL cell death (**Figure 5**). Furthermore, other biological function experiments indicated that SIRT1 may inhibit the migration and invasion of B-ALL by negatively regulating adhesion molecules and matrix metalloproteinase (**Figure 6**). SMAD4 participates in tumor metastasis and infiltration by transcriptionally regulating transforming growth factor beta (TGF-β) signaling pathway, such as MMP7 and N-cadherin (33), and was enriched in the PPI network we constructed (**Figure 4**). SIRT1 has been reported to deacetylate SMAD4 and repress the expression of its target genes (34), which implies that SIRT1 may be involved in the invasion of B-ALL by deacetylating SMAD4.

This study has several limitations. First, our findings are mainly based on the TARGET database, which means that a bigger sample size including diverse ethnic populations would be needed to confirm the predictive results of the SIRT family. Second, functional experiments were conducted only in cell line models, and the detailed molecular mechanisms require further exploration. Finally, the lack of direct experimental validation in patient samples or patient-derived xenograft models is crucial for fully confirming the translational relevance of our results.

## Conclusion

SIRT1 displays potential as diagnostic or prognostic biomarkers in childhood B-ALL. Further analysis showed that SIRT1 decreased vincristine resistance and invasion of B-ALL cell lines. Some inconsistent results were observed between this study and other types of leukemia highlighting the context-dependent impact of SIRT1, and further research is warranted to confirm the findings of SIRT1 in childhood B-ALL.

## Data Availability

The original contributions presented in the study are included in the article/**Supplementary Material**. Further inquiries can be directed to the corresponding authors.

## Supplementary material

Supplementary Figure 1 Gene expression analysis of SIRT family in acute lymphoblastic leukemia (ALL). **(A)** SIRT family mRNA expression levels in cancer and normal blood samples in ALL from the Therapeutically Applicable Research To Generate Effective Treatments (TARGET) database and the Genotype Tissue-Expression (GTEx) databases. **(B-E)** The receiver-operating characteristic (ROC) curve showed the high-expression specificity of SIRT1, SIRT3, SIRT4, and SIRT7 in ALL in the GTEx and TARGET databases. **(F)** The ROC curve showed the high-expression specificity of SIRT5 in ALL relapse in the TARGET databases. AUC, the area under the curve. * P < 0.05; ** P < 0.01; *** P < 0.001.

Supplementary Figure 2 Survival analysis and prediction model for event-free survival in ALL. **(A-G)** Kaplan-Meier analysis of SIRT family mRNA expression in ALL for event-free survival. **(H)** The nomogram was established to predict the 1-, 2- 3-, 5-, and 10-year event-free survival rate of patients with ALL. The predictive performance of the model on event-free survival was assessed by the C-Index. **(I)** Calibration plots were generated to compare the predicted event-free survival with the actual event-free survival at 1-, 2- 3-, 5-, and 10 years in ALL.

Supplementary Figure 3 Survival analysis and prediction model for overall survival (OS) in ALL. **(A-G)** Kaplan-Meier analysis of SIRT family mRNA expression in ALL for OS. **(H)** The nomogram was built to predict the 1-, 2- 3-, 5-, and 10-year OS of patients with ALL. The predictive performance of the nomogram was estimated using the C-Index. **(I)** Calibration plots were established to compare the predicted OS with the actual OS at 1-, 2- 3-, 5-, and 10 years in ALL.

Supplementary Figure 4 Copy number variation (CNV) and DNA methylation analysis of the SIRT family in ALL. **(A-G)** The relationship between SIRT family mRNA expression and CNV subtypes (normal and amplification copy) in ALL; **(H)** Correlation between DNA methylation and mRNA expression of SIRT family. Purple points represent a negative correlation and red points represent a positive correlation.
