# Supplementary material for: The integrated analysis of SIRT family expression, prognostic value, and potential implications in childhood acute lymphoblastic leukemia
Source: Front Oncol. 2025 Oct 15;15:1685249. doi: 10.3389/fonc.2025.1685249 (PMC12568422; doi:10.3389/fonc.2025.1685249)

Supplemental Information

Table S1. Demographic details in TARGET ALL cohort

Characteristic	Number of cases (%)	
Patients	480 (100)	
Gender	Female	169 (35.2)
	Male	311 (64.8)
Relapse	No	306 (63.8)
	Yes	156 (32.5)
	Unknown	18 (3.8)
CNS involvement grade	CNS1	355 (74.1)
	CNS2	102 (21.3)
	CNS3	22 (4.6)
MRD (Day 29)	0	283 (59.0)
	>0	191 (39.8)
	Unknown	6 (1.3)
Down's Syndrome	No	475 (99.0)
	Yes	2 (0.4)
	Unknown	3 (0.6)
Cell of origin	B precursor ALL	210 (43.8)
	T cell ALL	265 (55.2)
	other	5 (1.0)
Cytogenetics	ETV6-RUNX1 fusion gene	13
	Trisomy 4/10	20
	MLL Status	15
	TCF3-PBX1 fusion gene	16
	BCR-ABL1 fusion gene	5

CNS: Central nervous system, MRD: Minimal residual disease，ALL: acute lymphoblastic leukemia.

Table S2. The primers for the real-time quantitative polymerase chain reaction.

Primers	Species	Sequence (5' to 3')
GAPDH-F	Human	ACCCACTCCTCCACCTTTGAC
GAPDH-R	Human	TCCACCACCCTGTTGCTGTAG
MMP2-F	Human	ACCTACACCAAGAACTTCCGTCTG
MMP2-R	Human	TGCCAAGGTCAATGTCAGGAGAG
MMP9-F	Human	TGGTCCTGGTGCTCCTGGTG
MMP9-R	Human	TGCCTGTCGGTGAGATTGGTTC
E-cadherin-F	Human	ATTCTGCTGCTCTTGCTGTTTCTTC
E-cadherin-R	Human	CTCTTCTCCGCCTCCTTCTTCATC
N-cadherin-F	Human	GCGTGTGAAGGTTTGCCAGTG
N-cadherin-R	Human	CAGCACAAGGATAAGCAGGATGATG

CDK4 F	Human	CTGAAATTGGTGTCGGTGCCTATG
CDK4 R	Human	ACGAACTGTGCTGATGGGAAGG
cyclin D1 F	Human	CATGCTGAAGGCGGAGGAGAC
cyclin D1 R	Human	CCAGGTGGCGACGATCTTCC

F: forward primer; R: reverse primer.

Table S3. The diagnostic value of SIRT family gene expression for ALL

Gene name	Specificity(%)	Sensitivity(%)	cut-off value	AUC(%)	P value
**SIRT1**	98.5	98.1	2.7	**99.6**	**4.98E-129**
SIRT2	48.7	64.8	4.7	54.3	0.037
**SIRT3**	44.5	93.1	2.2	**75.0**	**3.59E-34**
**SIRT4**	83.4	83.1	0.4	**89.5**	**2.41E-82**
SIRT5	57.0	75.6	2.2	64.5	1.90E-12
SIRT6	39.5	88.5	3.0	60.7	1.97E-07
**SIRT7**	59.3	89.2	4.2	**76.3**	**1.14E-37**

ALL: acute lymphoblastic leukemia, AUC: the area under the curve.

Table S4. The diagnostic value of SIRT family gene expression for ALL relapse

Gene name	Specificity(%)	Sensitivity(%)	cut-off value	AUC(%)	P value
SIRT1	96.7	12.2	3.4	54.5	0.110
SIRT2	57.5	73.1	4.4	65.8	2.72E-08
SIRT3	79.4	34.0	2.6	55.8	0.041
SIRT4	85.0	51.3	0.6	68.5	7.39E-11
**SIRT5**	65.7	69.2	2.7	**70.8**	**2.73E-13**
SIRT6	86.3	26.9	4.1	54.3	0.130
SIRT7	62.4	59.6	3.3	61.5	5.048E-05

ALL: acute lymphoblastic leukemia, AUC: the area under the curve.

Table S5. Univariate and multivariate analysis of prognostic parameters in the childhood ALL for event-free survival.

	Univariate analysis		Multivariate analysis	
Variable	P-value	HR (95% CI)	P-value	HR (95% CI)
SIRT1 expression	**0.007**	0.801 (0.682, 0.941)	**0.004**	0.647 (0.479, 0.873)
SIRT2 expression	**4.70E-07**	1.760 (1.413, 2.193)	0.275	0.738 (0.427, 1.274)
SIRT3 expression	**0.032**	0.783 (0.626, 0.979)	0.115	0.655 (0.387, 1.108)
SIRT4 expression	**2.72E-09**	0.357 (0.254, 0.501)	0.929	1.022 (0.634, 1.648)
SIRT5 expression	**1.94E-17**	0.458 (0.382, 0.548)	0.173	0.759 (0.510, 1.128)
SIRT6 expression	0.056	1.316 (0.993, 1.744)		
SIRT7 expression	**2.41E-04**	0.647 (0.512, 0.816)	0.442	1.264 (0.696, 2.293)

Gender	**0.001**	0.589 (0.430, 0.806)	0.424	1.224 (0.747, 2.005)
WBC at diagnosis	0.068	0.999 (0.998, 1.000)		
MRD (Day 29)	**2.53E-05**	1.979 (1.440, 2.718)	0.821	0.942 (0.559, 1.585)
ETV6-RUNX1 fusion gene status	**0.002**	2.587 (1.424, 4.698)	0.712	0.842 (0.338, 2.096)
Trisomy 4/10 status	0.086	1.715 (0.926, 3.177)		
MLL status	0.262	0.520 (0.166, 1.632)		
TCF3-PBX1 fusion gene status	**5.59E-18**	13.322 (7.404, 23.970)	**9.55E-06**	5.092 (2.477, 10.469)
BCR-ABL1 fusion gene status	0.100	3.223 (0.798, 13.015)		
BMA blasts (Day 29)	**0.016**	1.094 (1.017, 1.176)	0.346	1.049 (0.949, 1.160)
Down's Syndrome	**0.006**	7.083 (1.741, 28.823)	0.499	1.709 (0.361, 8.086)
DNA index	**0.023**	3.791 (1.200, 11.976)	0.190	0.101 (0.003, 3.109)
Cell of origin	**1.89E-25**	0.081 (0.051, 0.130)	**5.67E-11**	0.017 (0.005, 0.057)

CI, confidence interval; HR, hazard ratio.

Table S6. Univariate and multivariate analysis of prognostic parameters in the childhood ALL for overall survival.

Variable	Univariate analysis P-value	Univariate analysis HR (95% CI)	Multivariate analysis P-value	Multivariate analysis HR (95% CI)
SIRT1 expression	**0.008**	0.773 (0.639, 0.936)	**0.007**	0.674 (0.505, 0.900)
SIRT2 expression	**6.42E-05**	1.707 (1.313, 2.218)	0.905	0.966 (0.546, 1.708)
SIRT3 expression	0.090	0.795 (0.610, 1.036)		
SIRT4 expression	**2.43E-05**	0.434 (0.294, 0.639)	0.519	1.207 (0.682, 2.136)
SIRT5 expression	**2.65E-12**	0.471 (0.382, 0.582)	**0.001**	0.503 (0.331, 0.764)
SIRT6 expression	**0.005**	1.612 (1.154, 2.251)	0.156	1.424 (0.874, 2.318)
SIRT7 expression	**0.002**	0.637 (0.482, 0.842)	0.082	2.065 (0.911, 4.678)
Gender	0.194	0.778 (0.533, 1.136)		
Relapse	**2.40E-08**	273.159 (38.076, 1959.653)	0.773	345745.413 (0, 1.78E+43)
WBC at diagnosis	0.225	0.999 (0.998, 1.000)		
MRD (Day 29)	**0.001**	1.947 (1.335, 2.841)	0.765	0.918 (0.523, 1.611)
Bone marrow site of relapse	**1.74E-26**	14.156 (8.692, 23.054)	0.175	2.381 (0.680, 8.336)
CNS site of relapse	**1.03E-06**	3.153 (1.989, 4.998)	0.039	0.442 (0.204, 0.959)
Testes site of relapse	0.141	2.87 (0.706, 11.668)		

Other sites of relapse	0.940	1.045 (0.331, 3.299)		
ETV6-RUNX1 fusion gene status	0.954	1.027 (0.417, 2.529)		
Trisomy 4/10 status	0.836	0.909 (0.369, 2.240)		
MLL status	0.411	0.555 (0.137, 2.255)		
TCF3-PBX1 fusion gene status	**1.15E-17**	12.968 (7.211, 23.322)	**0.001**	3.040 (1.562, 5.915)
BCR-ABL1 fusion gene status	0.054	3.100 (0.980, 9.809)		
BMA blasts (Day 29)	**0.014**	1.107 (1.021, 1.199)	0.256	1.057 (0.961, 1.163)
Down's Syndrome	**0.001**	10.244 (2.489, 42.167)	0.054	4.761 (0.974, 23.283)
DNA index	0.962	0.952 (0.124, 7.309)		
Cell of origin	**2.28E-15**	0.102 (0.058, 0.1800)	0.069	2.891 (0.921, 9.074)

CI, confidence interval; HR, hazard ratio.

Table S7. Gene set enrichment analysis (GSEA) of SIRT1 in B-ALL

Ontology	ID	Description	set Size	ES	NES	P value	FDR
BP	GO:0050684	regulation of mRNA processing	130	0.578303	1.79888	1.00E-10	1.53E-08
BP	GO:1903311	regulation of mRNA metabolic process	299	0.539272	1.72644	1.00E-10	1.53E-08
BP	GO:0006913	nucleocytoplasmic transport	330	0.513816	1.65061	1.00E-10	1.53E-08
BP	GO:0051169	nuclear transport	330	0.513816	1.65061	1.00E-10	1.53E-08
BP	GO:0008380	RNA splicing	444	0.495855	1.60551	1.00E-10	1.53E-08
BP	GO:0000375	RNA splicing, via transesterification reactions	302	0.493767	1.58133	1.00E-10	1.53E-08
BP	GO:0000377	RNA splicing, via transesterification reactions with bulged adenosine as nucleophile	298	0.492264	1.57589	1.00E-10	1.53E-08
BP	GO:0000398	mRNA splicing, via spliceosome	298	0.492264	1.57589	1.00E-10	1.53E-08
BP	GO:0043161	proteasome-mediated ubiquitin-dependent protein catabolic process	383	0.448716	1.44838	2.80E-09	3.43E-07
BP	GO:0000288	nuclear-transcribed mRNA catabolic process, deadenylation-dependent decay	67	0.561808	1.68007	9.55E-06	0.000471
CC	GO:0016607	nuclear speck	413	0.542661	1.75473	1.00E-10	1.53E-08
CC	GO:0035770	ribonucleoprotein granule	270	0.468668	1.49708	7.17E-09	8.29E-07
CC	GO:0036464	cytoplasmic ribonucleoprotein granule	253	0.474553	1.51008	8.85E-09	9.94E-07
CC	GO:0010494	cytoplasmic stress granule	92	0.568681	1.73915	5.52E-08	5.13E-06
CC	GO:0005681	spliceosomal complex	200	0.47476	1.49965	6.77E-07	4.68E-05
CC	GO:1902493	acetyltransferase complex	103	0.533062	1.63686	1.43E-06	8.83E-05
CC	GO:0031248	protein acetyltransferase complex	102	0.529711	1.62525	1.81E-06	0.000106
CC	GO:0008287	protein serine/threonine phosphatase complex	55	0.565873	1.668	4.38E-05	0.001672
CC	GO:1903293	phosphatase complex	55	0.565873	1.668	4.38E-05	0.001672
MF	GO:0003724	RNA helicase activity	73	0.64552	1.94473	1.00E-10	1.53E-08
MF	GO:0004386	helicase activity	150	0.598126	1.86872	1.00E-10	1.53E-08
MF	GO:0019783	ubiquitin-like protein peptidase activity	116	0.587023	1.81372	1.00E-10	1.53E-08
MF	GO:0016887	ATP hydrolysis activity	398	0.488178	1.57647	1.00E-10	1.53E-08
MF	GO:0008186	ATP-dependent activity, acting on RNA	75	0.638247	1.92638	1.70E-10	2.50E-08

MF	GO:0042393	histone binding	246	0.484012	1.53885	3.95E-09	4.70E-07
MF	GO:0070063	RNA polymerase binding	57	0.642178	1.89938	3.65E-08	3.56E-06
MF	GO:0043175	RNA polymerase core enzyme binding	41	0.67884	1.95486	1.25E-07	1.08E-05
MF	GO:0000993	RNA polymerase II complex binding	36	0.685639	1.94819	3.65E-07	2.81E-05
MF	GO:0140297	DNA-binding transcription factor binding	474	0.395634	1.28313	7.95E-05	0.002626
KEGG	hsa03013	Nucleocytoplasmic transport	108	0.598426	1.821861	1.00E-10	3.05E-08
KEGG	hsa03015	mRNA surveillance pathway	97	0.571635	1.730039	6.66E-09	1.02E-06
KEGG	hsa03083	Polycomb repressive complex	82	0.57567	1.716903	2.27E-07	2.31E-05
KEGG	hsa03040	Spliceosome	129	0.461493	1.421378	0.000235	0.005118
KEGG	hsa04141	Protein processing in endoplasmic reticulum	167	0.442595	1.38536	0.000187	0.005118
KEGG	hsa04068	FoxO signaling pathway	130	0.462527	1.423689	0.000276	0.005611
KEGG	hsa04110	Cell cycle	158	0.432259	1.346609	0.00092	0.012206
KEGG	hsa04350	TGF-beta signaling pathway	108	0.441372	1.343723	0.003055	0.026626

BP, biological process; CC, cellular component; MF, molecular function; ES, Enrichment score.

Figure S1

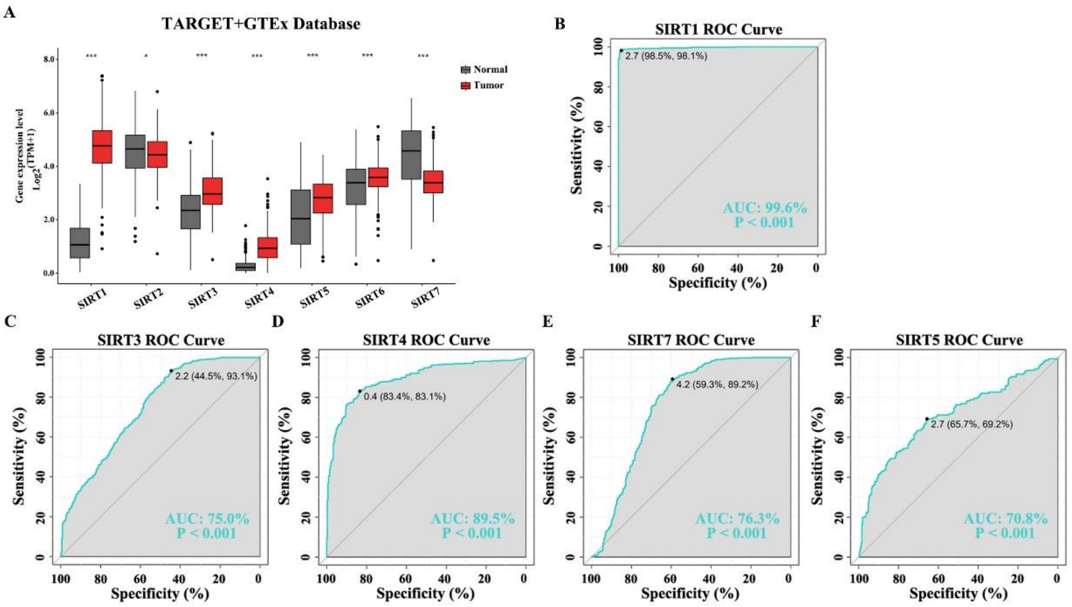

Figure S2

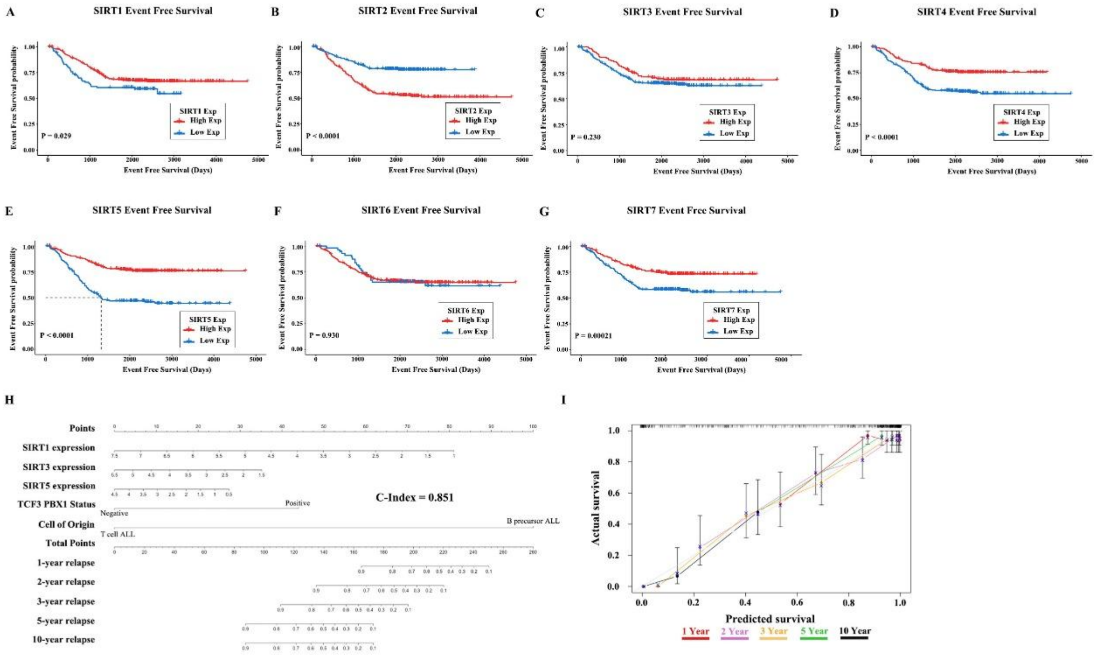

Figure S3

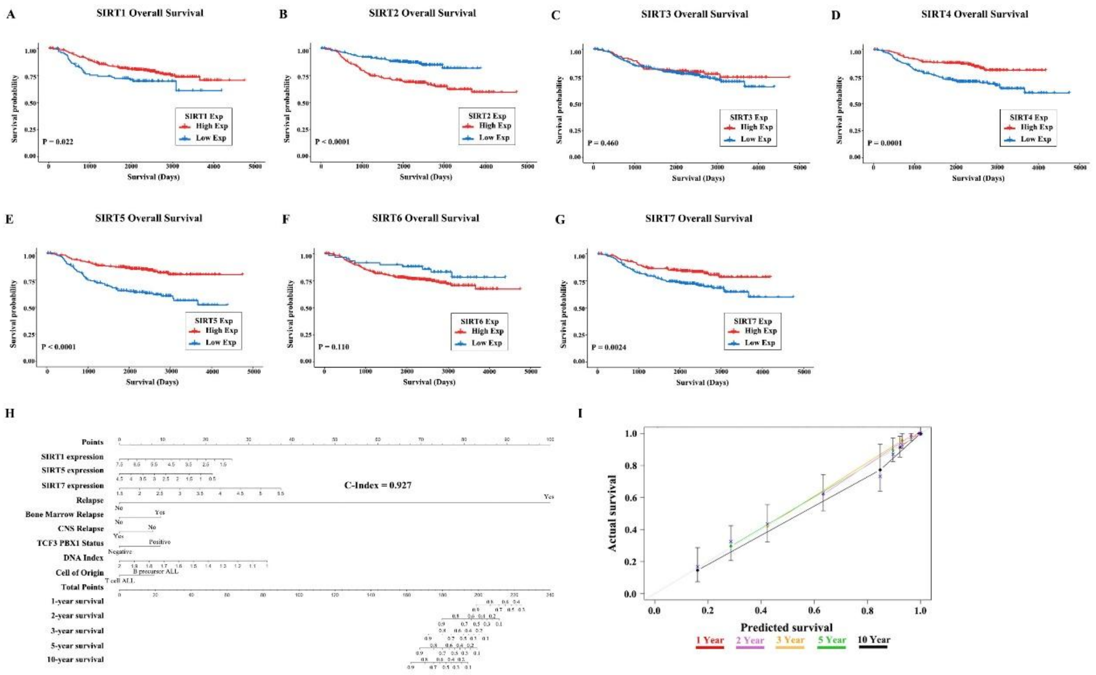

Figure S4

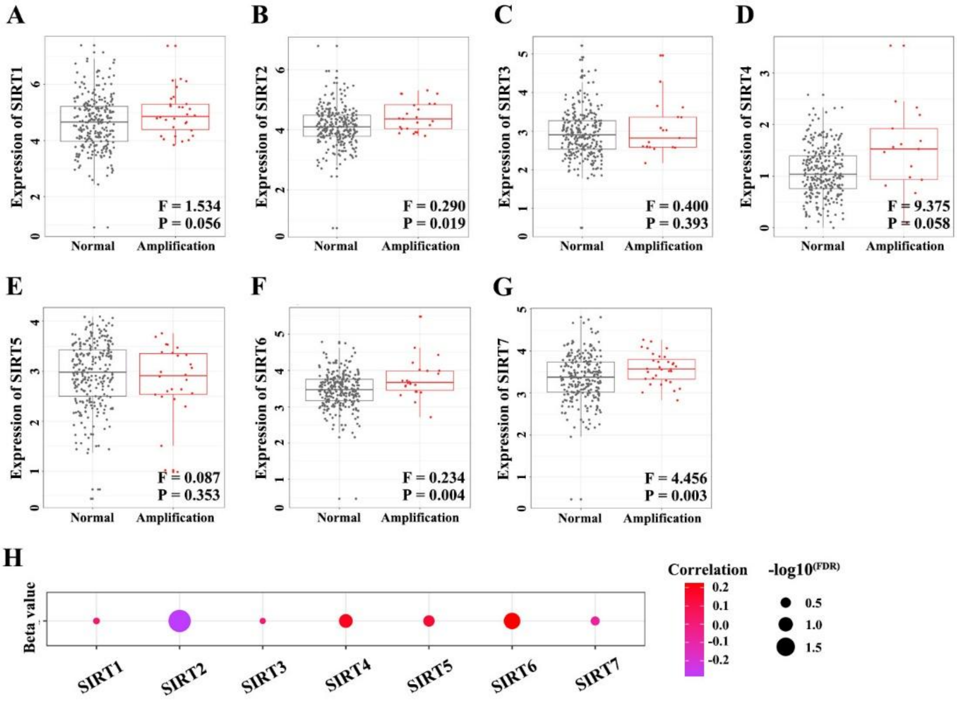

WB original data

Figure 5A HALM6

SIRT1

Control **SRT2104**

140KD

100KD

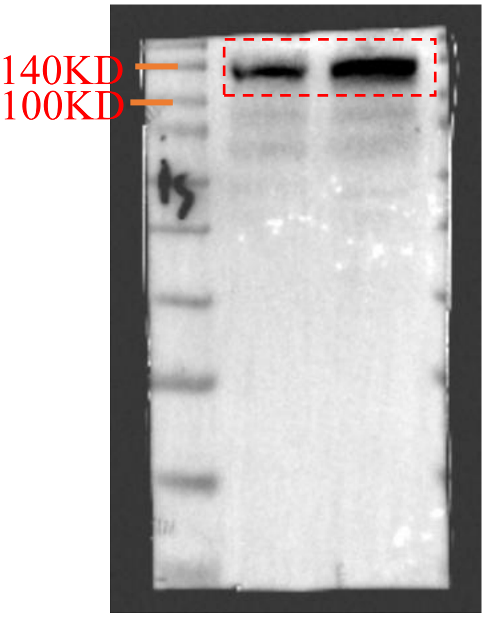

Bcl2

Control **SRT2104**

25KD

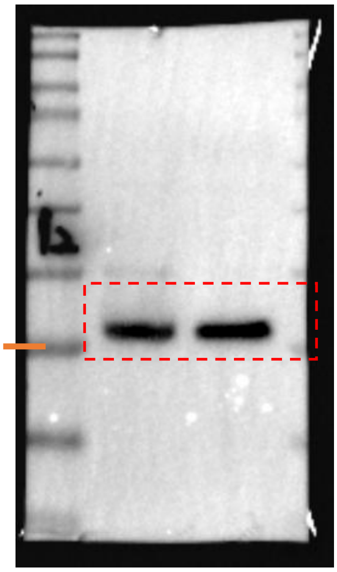

Bax

Control **SRT2104**

25KD

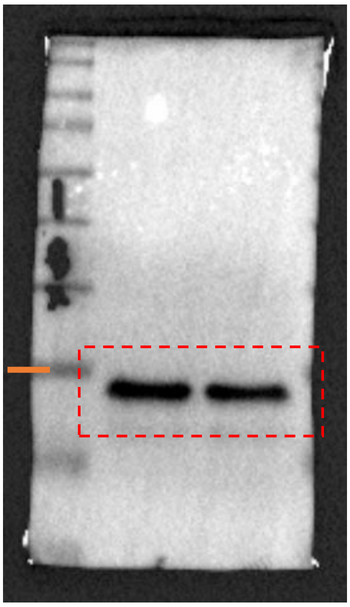

β-tubulin

Control **SRT2104**

60KD

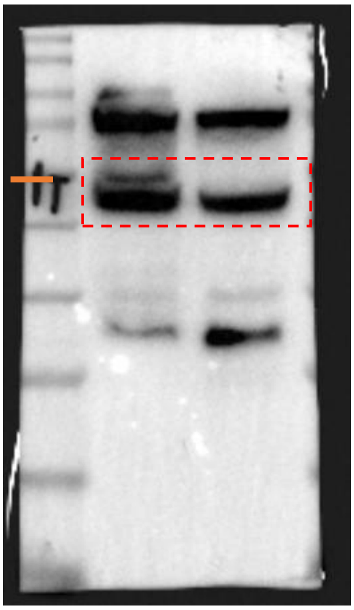

Figure 5B REH

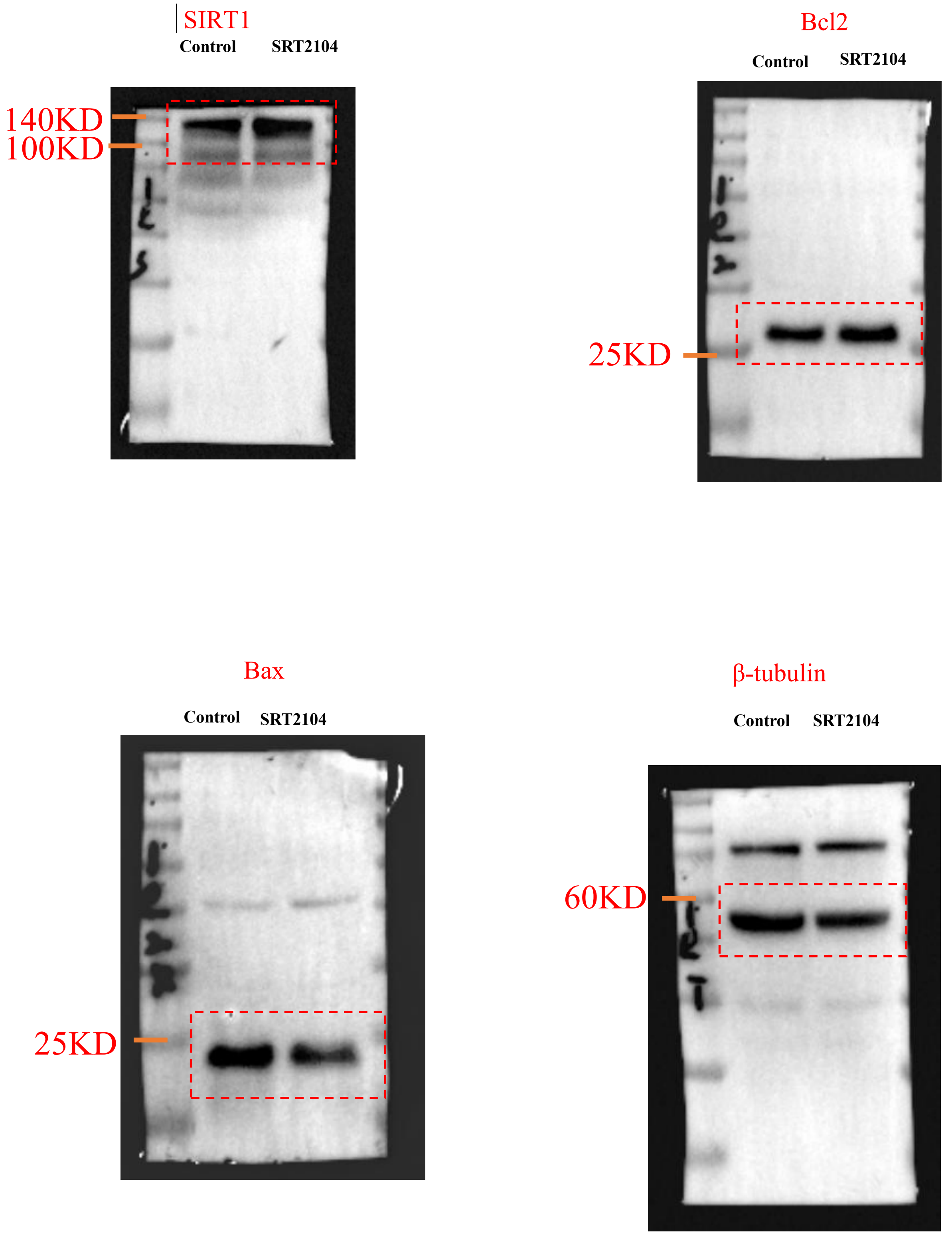

Supplement: Supplementary Figure 1 — Gene expression analysis of SIRT family in acute lymphoblastic leukemia (ALL). (A) SIRT family mRNA expression levels in cancer and normal blood samples in ALL from the Therapeutically Applicable Research To Generate Effective Treatments (TARGET) database and the Genotype Tissue-Expression (GTEx) databases. (B-E) The receiver-operating characteristic (ROC) curve showed the high-expression specificity of SIRT1, SIRT3, SIRT4, and SIRT7 in ALL in the GTEx and TARGET databases. (F) The ROC curve showed the high-expression specificity of SIRT5 in ALL relapse in the TARGET databases. AUC, the area under the curve. * P < 0.05; ** P < 0.01; *** P < 0.001. [file DataSheet1.pdf]
